# Evaluation of the Oral Microbiome in Patients with Alström and Bardet-Biedl Syndromes and Their Heterozygous Family Members

**DOI:** 10.3390/microorganisms13112442

**Published:** 2025-10-24

**Authors:** Ewa Zmysłowska-Polakowska, Tomasz Płoszaj, Sebastian Skoczylas, Julia Grzybowska-Adamowicz, Aleksandra Palatyńska-Ulatowska, Monika Łukomska-Szymańska, Agnieszka Zmysłowska

**Affiliations:** 1 Department of Endodontics, Medical University of Lodz, 92-213 Lodz, Poland; aleksandra.palatynska-ulatowska@umed.lodz.pl; 2 Department of Clinical Genetics, Medical University of Lodz, 92-213 Lodz, Poland; tomasz.ploszaj@umed.lodz.pl (T.P.); sebastian.skoczylas@umed.lodz.pl (S.S.); julia.grzybowska-adamowicz@umed.lodz.pl (J.G.-A.); agnieszka.zmyslowska@umed.lodz.pl (A.Z.); 3 Department of General Dentistry, Medical University of Lodz, 92-213 Lodz, Poland; monika.lukomska-szymanska@umed.lodz.pl

**Keywords:** Alström syndrome, Bardet-Biedl syndrome, heterozygous carriers, oral microbiome, gene sequencing

## Abstract

Alström (ALMS) and Bardet-Biedl syndromes (BBS) are rare ciliopathies characterized by obesity and hyperglycemia that lead to type 2 diabetes, but also other disorders, including neurodegeneration. However, isolated clinical manifestations can be observed in carriers of heterozygous mutations in the *ALMS1* and *BBS* genes. Recently, the influence of oral bacteria on the presence of obesity, type 2 diabetes, and neurodegenerative processes has been widely discussed. The purpose of the research project was to analyze the profile of the microbiome in the oral cavity by sequencing the 16S rRNA gene in ALMS/BBS patients and carriers of causative variants in these genes. Oral mucosal swabs were taken from 8 ALMS/BBS patients, 24 family members, 20 obese patients, and 29 healthy individuals. *Streptococcus* (30.7%), *Haemophilus* (18.9%), and *Prevotella* (11%) were the most common bacteria in the study group. Comparison between groups showed a higher abundance of *Prevotella*, *Enterococcus*, *Eikenella*, *Capnocytophaga*, *Parvimonas*, *Selenomonas*, and *Corynobacterium*, and a lower abundance of *Lactobacillus* in the study group compared to other groups. The specific profile of the oral microbiome found in patients with variants in the *ALMS1* and *BBS* genes may enable the identification of the modulatory role of the oral microbiome in these disorders and point to new directions for additional therapy for these patients and heterozygous family members in the future.

## 1. Introduction

Alström syndrome (ALMS) and Bardet-Biedl syndrome (BBS) represent rare diseases inherited in an autosomal recessive manner classified as ciliopathies, which underlie their common pathological mechanism and make them monogenic diabetic syndromes [1,2]. The estimated prevalence of BBS is approximately 1:160,000 in northern European populations, while ALMS is even rarer, occurring in 1:500,000 individuals [3,4,5]. In both syndromes, obesity appears as the first clinical symptom, which, along with the progression of insulin resistance and metabolic disorders, leads to the development of type 2 diabetes in most patients [6,7,8]. Another common early symptom for patients with both ALMS and BBS is visual impairment, ultimately defined as progressive retinal degeneration [9].

In addition, patients with ALMS and BBS may have other ophthalmologic, cognitive, and neurologic disorders of neurodegenerative origin, but also renal, pulmonary, and cardiac abnormalities, as well as bone defects, liver dysfunction, low stature and endocrine disorders [9,10,11,12,13]. Specifically, ALMS patients often exhibit short stature, skin changes such as *acanthosis nigricans*, hypertension, atherosclerosis, cataracts, cardiomyopathy in approximately 60% of patients, hearing impairment and deafness, bronchial asthma and pulmonary symptoms (50%), chronic hepatitis with fatty liver (50%), chronic renal failure, gynecomastia, alopecia, menstrual disorders and urological disorders in women, hypergonadotropic hypogonadism in men, diabetes insipidus, and neurological disorders [9,10,14].

The symptoms in patients with BBS are very similar. However, this syndrome also involves abnormalities in the long bones of the limbs, mainly in the form of polydactyly, brachydactyly, and syndactyly. Patients with BBS often suffer from cognitive impairment and/or intellectual disability [11,12,15]. Clinical observations suggest that isolated clinical manifestations may also occur in carriers of heterozygous mutations in the *ALMS1* and *BBS* genes, i.e., in parents and siblings of patients with ALMS and BBS [2,16,17,18,19,20].

Although the genetic causes behind both syndromes have been extensively studied, evidence suggests that environmental factors may also influence the clinical manifestations and courses of the disease in individual patients. This may determine the diversity of clinical presentations among patients and different phenotypes present in members of the same families and in individuals who are carriers of the same causative genetic variant [21,22].

Among the potential environmental factors modulating the natural course of various chronic diseases is the contribution of oral bacteria, which has received considerable attention in recent years [23,24,25]. These articles described the importance of the protective tissue barrier in the oral cavity, as its disruption allows pathogenic microorganisms to migrate and influence the development of further disorders, including neurodegeneration [26,27]. Moreover, numerous findings highlight the combined influence of genetic and environmental factors, particularly specific bacterial species affecting the expression of specific genes, especially in conditions where periodontal disease is a contributing factor [24,28,29]. Finally, understanding the composition of the microbiome in patients with obesity and type 2 diabetes may translate into more effective treatment of these diseases [30,31].

Given the imprecise classification of some bacteria species in the microbiological studies used to date, it is reasonable to apply new methods to detect the microbial genome in the search for additional modulating mechanisms in patients with defined monogenic diabetes syndromes.

The goal of this study was to identify the microbial genome in buccal mucosal swabs from patients with Alström and Bardet-Biedl syndromes and their families, who are carriers of heterozygous mutations in the *ALMS1* and *BBS* genes, in comparison to obese patients (comparison group) and healthy controls.

## 2. Patients and Methods

### 2.1. Characteristics of Patients and Examination

The study group included 8 participants with genetically confirmed ALMS (2) or BBS (6) syndromes from 8 families, as previously described [21,22]. All these participants were overweight or obese. Additionally, the group included 24 heterozygous carriers of causative variants in the *ALMS1* and *BBS* genes from the same families (11 and 13 carriers, respectively), while the comparison group comprised 20 patients with simple obesity matched for BMI (Body Mass Index) (*p* = 0.989). The control group comprised 29 healthy individuals (BMI < 25 kg/m^2^ and without hyperglycemia, type 2 diabetes, retinal disorders, cardiomyopathy, renal dysfunction, or cirrhosis) matched to the other groups for age (*p* = 0.997) and gender (*p* = 0.507). Of the 24 heterozygous carriers of the causative variants, 14 individuals were overweight/obese and/or had type 2 diabetes. The exclusion criteria were the use of oral antibiotics and hormonal contraceptives within the last 2 months, as well as pregnancy.

Table 1 presents a detailed characterization of study participants.

All studied individuals underwent a precise dental examination of the oral cavity performed by two experienced dentists. The Oral Hygiene Index (OHI), according to *Greene and Vermillion*, which assesses the state of oral hygiene, was recorded. The OHI is the sum of the plaque and calculus index. The plaque score ranged from 0 (no plaque), to 1 (soft plaque or supragingival calculus that covers a minimum of 1/3 of the tooth surface), 2 (soft plaque or supragingival calculus that covers 1/3 to 2/3 of the tooth surface or isolated subgingival calculus around the tooth neck), and 3 (soft plaque or supragingival calculus coating more than 2/3 of the tooth surface or dense accumulation of subgingival calculus encircling the tooth neck) [32]. No participant in the study, reference, or control groups was diagnosed with periodontal disease.

To assess clinical gingival inflammation, all subjects were evaluated using the Löe and Silness gingival index (GI) and the bleeding on probing score (BOP%) [33]. The values did not exceed 0.2 and 10%, respectively, in any group. Moreover, there were no significant differences in GI (*p* = 0.407) and BOP% (*p* = 0.465) scores among the groups.

Next, non-invasive sampling of the buccal mucosa and buccal gingival margin in the region of the first lower permanent molar was performed in participants from the study, comparison, and control groups. We used a method of sampling the buccal mucosa and gingival area that, according to the literature, is optimal and recommended for assessing the microbiome, especially in the absence of periodontitis [34,35]. All study participants were asked to refrain from eating, smoking, and oral hygiene activities (brushing, flossing, and using mouthwash) for 12 h prior to sample collection, and from drinking for 1 h prior to the test.

The University Bioethics Committee at the Medical University in Lodz, Poland (RNN/216/23/KE) reviewed and approved the research protocol on 10 October 2023. Written informed consent for participation in the project was collected from patients and/or their parents. Collected samples intended for molecular analysis were transferred into sterile screw-cap vials and stored at −20 °C until further processing.

### 2.2. Molecular Analysis—DNA Isolation

Collected buccal swabs were first frozen. Bacterial DNA was then isolated from those samples using the Maxwell^®^ RSC Pathogen Total Nucleic Acid Kit catalog number: AS1890 (Promega, Madison, WI, USA). The isolated DNA was suspended in TE buffer and stored at −20 °C until PCR amplification. DNA concentration and purity were determined spectrophotometrically using a NanoPhotometer^®^ C40 (Implen, München, Germany).

### 2.3. Library Preparation and Sequencing

To assess the microbial community profiles, the 16S rRNA gene was sequenced. Initially, the V3/V4 variable regions of the genome were amplified following the protocol advised by Illumina (San Diego, CA, USA). PCR oligonucleotides with overhanging adapters compatible with Illumina Nextera indexes and sequencing adapters for paired-end sequencing were utilized (forward, TCGTCGGCAGCGTCAGATGTGTATAAGAGACAGCCTACGGGNGGCWGCAG; reverse, GTCTCGTGGGCTCGGAGATGTGTATAAGAGACAGGACTACHVGGGTATCTAATCC). The amplification of the gene fragments, averaging 464 base pairs in length, was performed using Kapa HiFi polymerase (Roche, Mannheim, Germany). The specificity of the PCR products was verified using agarose gel electrophoresis, followed by purification with AMPure XP magnetic beads (Beckman Coulter, CA, USA). Indexing reactions were also conducted using Kapa HiFi polymerase (Roche, Mannheim, Germany) with the Nextera XT dual-index set (Illumina, San Diego, CA, USA). Library concentrations were measured with the Qubit 2.0 system (Thermo Fisher Scientific, Waltham, MA, USA), and the libraries were pooled at equal concentrations. The prepared library was then sequenced on the MiSeq device (Illumina, San Diego, CA, USA) using the MiSeq Reagent Kit v3, 2x300 cycles.

### 2.4. NGS Data Processing

Sequencing data from the MiSeq platform were processed using a local instance of the Galaxy platform 20.01 [36]. The FASTQ Groomer tool was used to convert files to Sanger FASTQ encoding. Paired-end reads were then combined using the FLASH tool 1.2.11.4 [37], and the Trimmomatic 0.38.0 algorithm was applied to separate adapters and filter out low-quality reads (those below a quality score of Q20 and length of 220 bp) [38]. Operational Taxonomic Units (OTUs) were allocated using the Kraken 2 v.1.3.1 [39] algorithm with the Standard database, with filtering based on a classification confidence score threshold of 0.05.

Data were filtered to remove poor quality and non-informative data using a low count filter (minimum count of 4) and a low variance filter (inter-quartile range 10%). Data were then normalized using the total sum scaling (TSS) method. The OTU read counts for each taxonomic level were extracted and organized into tables. The relative abundance of each recognized bacterial taxon was then determined from these tables.

### 2.5. Data Analysis—Alpha and Beta Diversity

Microbiome composition diversity in the samples was assessed at the genus level using the MicrobiomeAnalyst 2.0 (update from 2 July 2024) [40]. To determine alpha diversity and statistical significance, Shannon algorithms, along with the ANOVA test, were employed. For beta diversity analysis, non-metric multidimensional scaling (NMDS) was used to visualize the data, with statistical significance assessed using the ANOSIM test.

### 2.6. Visualization of Data and Statistical Analysis

The normality distribution of the parameters was verified with the Shapiro–Wilk test. For comparisons of the BMI and OHI values, the Mann–Whitney U test was used. Gender distribution was compared using the chi-square test, and patients’ ages were compared using ANOVA. Correlations between parameters were calculated using Pearson’s correlation test. We conducted hierarchical clustering and heatmap analysis of the molecular data using Ward’s clustering algorithm and the Euclidean distance method. Differences in raw abundances between groups were visualized using stacked bar plots. The overall changes in the relative abundance of genera in single-factor analysis were assessed using the EdgeR method. Statistical significance was defined as a false discovery rate (FDR) below 0.05. The relevance or effect size of different genus abundance between groups was evaluated using the LEfSe (Linear discriminant analysis Effect Size) algorithm, with statistical significance defined as an FDR-adjusted *p*-value below 0.1.

## 3. Results

In the initial comparison, no statistically significant differences were observed between homozygous and heterozygous carriers of *ALMS1* and *BBS* gene variants, both in relation to the OHI (*p* = 0.323) and alpha diversity (*p* = 0.424). This justified combining these patients into a single study group to increase the statistical power of the study, given the rarity of these genetic syndromes.

### 3.1. Oral Hygiene Index (OHI) Values

**Figure 1:**
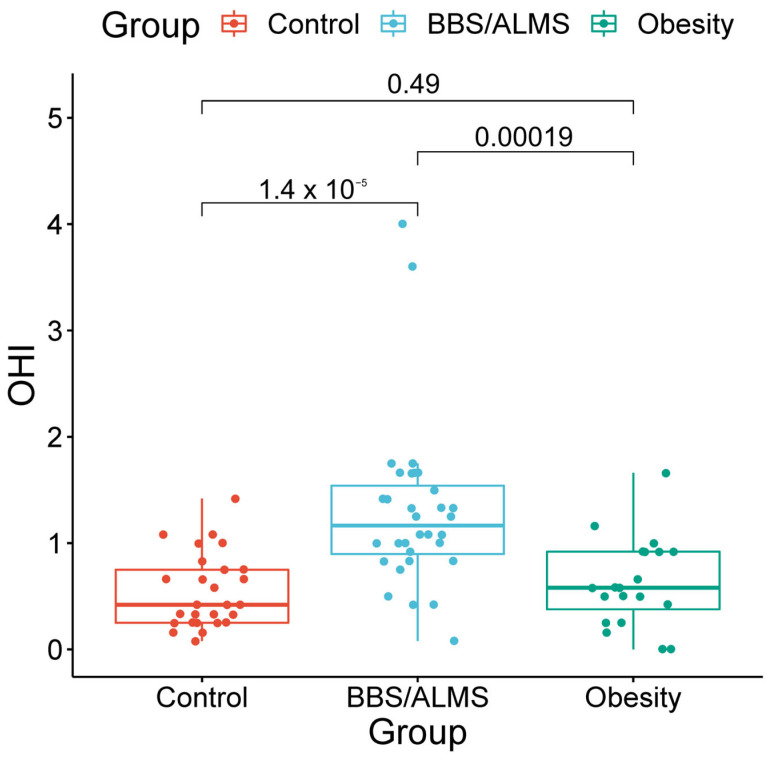
Box plots of the Oral Hygiene Index (OHI) with marked *p*-values for the *t*-test.

The Oral Hygiene Index (OHI) values differed between the groups. The highest OHI was observed in the study group (Me 1.25; IQR 0.62), which was significantly higher than in both the simple obesity group (Me 0.54; IQR 0.67; *p* < 0.001) and the healthy control group (Me 0.42; IQR 0.5; *p* < 0.00001). No significant difference in OHI values was found between the obese and healthy control groups (*p* = 0.490) (Figure 1).

In both the study group and the comparison and control groups, there was no significant correlation between the OHI and patient age (*p* respectively: 0.327; 0.229 and 0.190).

### 3.2. Oral Microbiome RNA Sequencing

**Figure 2:**
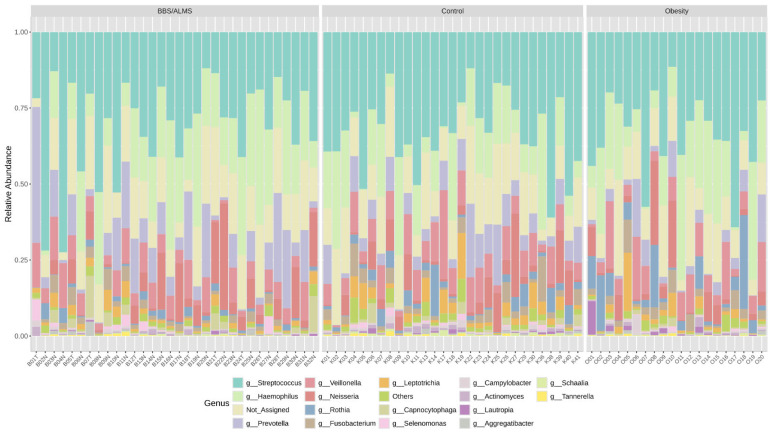
Taxonomic composition of the bacterial community at the genus level in the studied groups.

**Figure 3:**
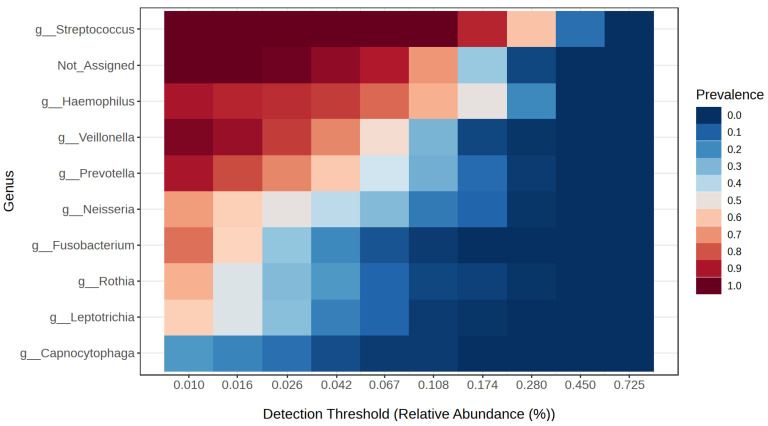
Core microbiome genera of bacteria in the studied groups.

Following the 16S rRNA gene sequencing, a total of 6,721,299 reads classified as bacteria were obtained. Each sample had an average of 83,124 reads, and, on average, 10.7% of the reads could not be assigned to any bacterial genus. Next-generation sequencing (NGS) identified Streptococcus (30.7%), Haemophilus (18.9%) and Prevotella (11%) as the most prevalent genera in the study group. In the obesity group, Streptococcus was the most common, accounting for 33.3%, followed by Haemophilus (18%) and Veillonella (8.3%). The control group was dominated by Streptococcus (33.1%), Haemophilus (14.1%), and Veillonella (8.7%). Figure 2 presents a detailed distinction of the types of bacteria found in all the studied groups. An overall analysis of the core microbiome is shown in Figure 3.

### 3.3. Diversity and Discriminant Analyses

**Figure 4:**
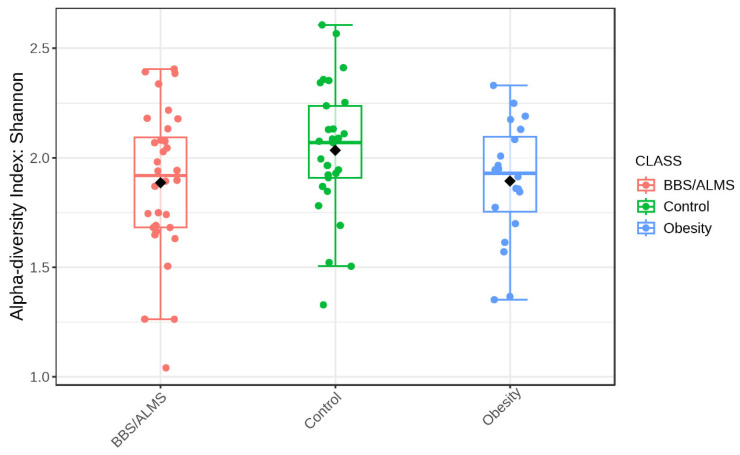
Box plot illustrating the overall measure of alpha-diversity across the studied groups based on the Shannon method at the bacterial genus level. The black dot indicates the average value. Statistical significance was evaluated by ANOVA, with an F-value of 2.0673 and a *p*-value of 0.1334.

**Figure 5:**
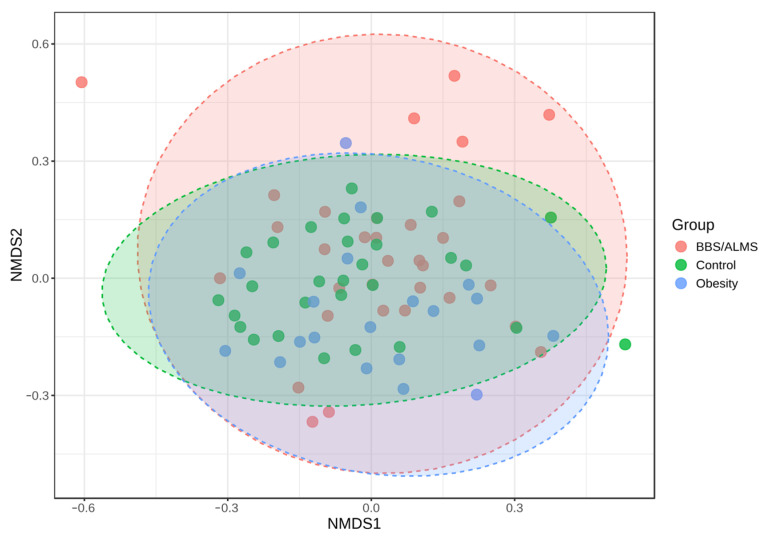
Non-metric multidimensional scaling (NMDS) analysis diagram based on the Bray-Curtis distance. Statistical significance was evaluated by ANOSIM, with a *p*-value < 0.031; [NMDS] Stress = 0.2384.

Analysis of alpha diversity using Shannon’s method indicated no statistically significant differences in diversity between the studied groups. However, there was a trend suggesting that the control group had the highest average alpha diversity compared to the other two studied groups, *p* = 0.133 (Figure 4). However, beta diversity, an analysis that indicates differences in the composition of bacterial genera, suggests that the BBS/ALMS group stands out from the other studied groups, *p* < 0.031 (Figure 5).

**Figure 6:**
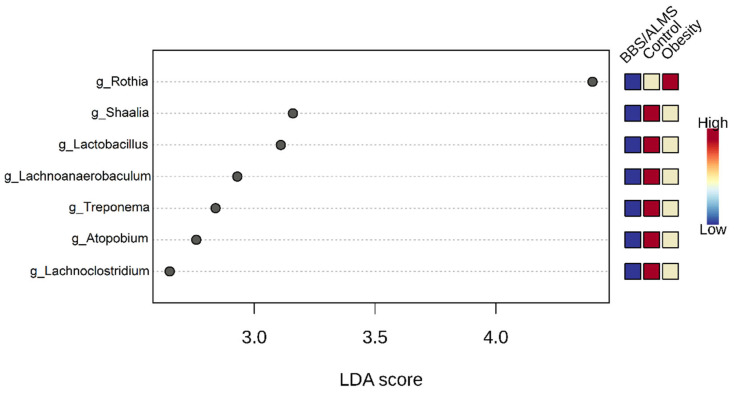
Linear discriminant analysis Effect Size (LEfSe) analysis of taxonomic biomarkers of oral microbiota. The analysis determined the bacterial genera with the most differential abundance. The top seven genera are shown with *p*-value < 0.015. Statistical values were evaluated by the Kruskal-Wallis rank sum test.

Interestingly, the LEfSe analysis identified the seven most differentiating bacterial genera, including the three main ones: Rothia, Shaalia and Lactobacillus, for which the Linear Discriminant Analysis (LDA) score was above 3 (*p* < 0.015) (Figure 6).

### 3.4. Univariate Analysis

**Figure 7:**
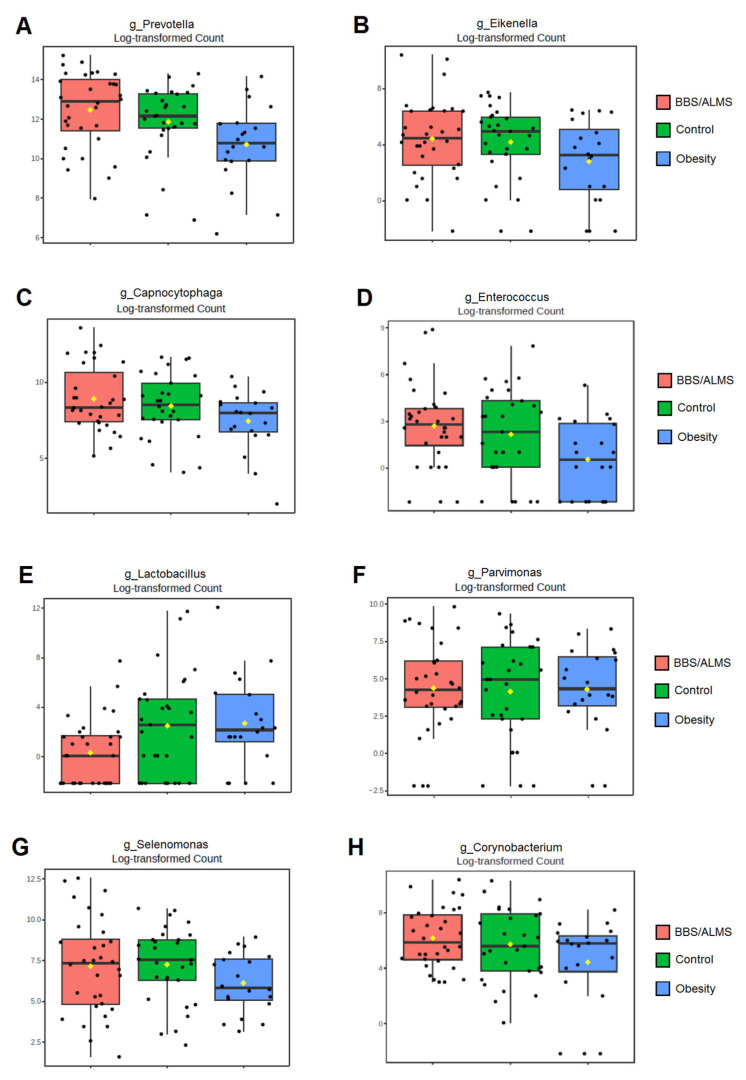
Box plots of univariate analysis for: (**A**)—*Prevotella*; (**B**)—*Eikenella*; (**C**)—*Capnocytophaga*; (**D**)—*Enterococcus*; (**E**)—*Lactobacillus*; (**F**)—*Parvimonas*; (**G**)—*Selenomonas*; (**H**)—*Corynobacterium;* horizontal line inside each box represents the median; yellow dot indicates the mean; black dots represent individual data points.

Univariate analysis showed that eight bacterial genera were significantly different in the study group compared to both the obese and healthy groups (Figure 7). The genera Prevotella and Enterococcus were considerably more abundant in the BBS/ALMS group than in the group with obesity (*p* = 0.00006 and 0.00007, respectively) and the control group (*p* = 0.0034 and 0.0088, respectively) (Figure 7A,D). In contrast, Eikenella, Capnocytophaga, Parvimonas, and Selenomonas were more abundant in the study group than in the obese group (*p* = 0.000006, 0.00004, 0.0044, and 0.000002, respectively) but less abundant than in the healthy group (*p* = 0.00001, 0.0001, 0.00016, and 0.0000015, respectively) (Figure 7B,C,F,G). Furthermore, Corynobacterium was more abundant in the BBS/ALMS group than in the healthy group (*p* = 0.00824), but less abundant than in the obese group (*p* = 0.00031) (Figure 7H). Lactobacillus abundance was lowest in the study group compared to both other groups (*p* = 0.00087 and 0.0000003, respectively) (Figure 7E).

## 4. Discussion

This study represents the first evaluation of the oral microbiome in patients with Alström and Bardet-Biedl syndromes and heterozygous carriers of causative variants in the *ALMS1* and *BBS* genes. Importantly, although none of the subjects had periodontitis, patients in the study group had the highest OHI scores, indicating inadequate oral hygiene. Due to neurodevelopmental and intellectual disorders, our patients have and, unfortunately, will continue to have poorer hygiene, as described in many genetic syndromes, including ciliopathies [41,42,43,44,45]. Recently, higher OHI values were described in patients with poor metabolic control of type 2 diabetes and in patients with type 2 diabetes and obesity [46,47].

In the study group, the most prevalent oral bacteria were *Streptococcus*, *Haemophilus* and *Prevotella*. An increased abundance of the genus *Prevotella* has previously been reported in studies investigating changes in the oral microbiome of patients with type 2 diabetes [48] and overweight or obese individuals [49]. While no statistically significant distinctions in alpha diversity were found between groups, beta diversity analysis showed differences in the composition of bacterial types between groups. Almeida-Santos et al. reported no differences in alpha and beta diversity between healthy individuals and patients with type 2 diabetes [50].

However, further analysis identified eight bacterial genera that differentiate patients carrying at least 1 causative variant in the *ALMS* and *BBS* genes from the comparison and control groups. The higher abundances of *Eikenella*, *Capnocytophaga* and *Selenomonas* were reported in the BBS/ALMS group compared to patients with simple obesity, which may correspond with the reported increase in these bacteria in patients with diabetes [51,52,53]. A diversity of bacterial types has already been observed in patients with type 2 diabetes compared to normoglycemic individuals [54,55].

The relationships between diabetes and oral microbiota are complex. Periodontal disease is known to be influenced by type 2 diabetes [56,57,58]. On the other hand, it may worsen glycemic control [57]. Moreover, high levels of certain bacteria, such as *Porphyromonas*, can exacerbate insulin resistance [58]. Alterations in the oral microbiome have also been found to affect metabolic control of diabetes as measured by HbA1c levels in patients with type 1 diabetes [59,60].

Goodson et al. associated an increased presence of *Selenomonas* sp. and *Prevotella* sp. with being overweight, while our study reported an even higher abundance of *Selenomonas* and *Prevotella* genera in the ALMS/BBS group compared to participants with simple obesity [49]. Other studies have linked the genera *Veillonella*, *Oribacterium*, and *Soonwooa* [61] as well as *Firmicutes* and *Actinobacteria* [62] with obesity. Changes in oral microbiome composition appear to be associated with obesity development in multiple mechanisms [63], as well as different taste perceptions [64,65]. Other systemic mechanisms may involve changes in the composition of gut bacteria. This may lead to changes in adipose tissue function, inflammation along the oral–blood axis, and feeding behavior via the gut–brain axis [63,66].

Patients with ALMS and BBS may also present neurologic disorders of neurodegenerative origin [9,10,11,12,13]. Changes in gut and oral microbiome were reported to have a significant role in the development of psychiatric and neurodegenerative disorders [26,27,67]. The changes in the oral microbiome may lead to periodontitis, a known severe risk factor for Alzheimer’s disease. Alzheimer’s-related brain immunity and neuroinflammation are thought to be caused by prolonged exposure to various microorganisms and/or their toxins, rather than by any specific bacteria [67].

Additionally, patients with ALMS and BBS were observed to have dental anomalies such as abnormalities in tooth number, shape, and size [68,69,70,71]. Patients with BBS are also more prone to oral infections, such as periodontal disease and dental caries, and they may experience drug-induced gingival hyperplasia [68,70]. Meanwhile, gingivitis and discolored enamel were reported in ALMS patients [72].

Moreover, in our study, the abundance of *Lactobacillus* genera was the lowest in the study group. Interestingly, the association between dysbiosis and periodontal disease has already been found in patients with diabetes [24,29]. Regarding specific beneficial genera, the therapeutic use of *Lactobacillus* sp. in periodontitis associated with type 2 diabetes is established, operating through various mechanisms [73]. Furthermore, *Lactobacillus* sp. may also be helpful in reducing body weight in overweight and obese patients [74].

The relatively small sample size represents a limitation of our study, reflecting the rarity of ALMS and BBS [9,12]. Consequently, the data should be considered preliminary. Furthermore, the molecular analysis of *ALMS1* and *BBS* genes was not performed in the comparison and control groups. However, clinical examination and family histories of overweight, obesity, type 2 diabetes, and additional disorders (no ophthalmic, hepatic, or renal abnormalities, or cardiomyopathy) conducted by an experienced clinical geneticist did not indicate suspected monogenic diabetes syndromes in these individuals.

In conclusion, evaluating the oral microbiome of patients with Alström and Bardet-Biedl syndromes and heterozygous carriers of causative variants in the *ALMS1* and *BBS* genes compared to patients with simple obesity and healthy subjects revealed a distinctive oral bacterial profile in ALMS/BBS patients. Further research is needed to link the oral bacterial profile with the prevalence and severity of clinical symptoms in both ALMS/BBS patients and symptomatic members of their families carrying the genetic defect. Such studies will enable future evaluation of the modulating role of oral microflora in these disorders and may inform therapeutic strategies for these patients and their heterozygous family members.

## Figures and Tables

**Table 1:** Characteristics of the studied groups.

Group	Gender (%)	BMI (kg/m^2^) Median (IQR)	Age (years) Mean ± SD
BBS/ALMS (study group; n = 32)	60% F; 40% M	29.7 (12.1)	30.3 ± 15.8
Obesity (comparison group; n = 20)	66.7% F; 33.3% M	37.8 (17.4)	30.6 ± 3.9
Control group; n = 29	73.3% F; 26.7% M	22.3 (5.3)	31.0 ± 13.9

F—female, M—male, SD—standard deviation, BMI—body mass index, and IQR—interquartile range.

## Data Availability

The original contributions presented in this study are included in the article. Further inquiries can be directed to the corresponding author.

## Abbreviations

The following abbreviations are used in this manuscript:

ALMS	Alström syndrome
ANOVA	Analysis of Variance
BBS	Bardet-Biedl syndrome
BMI	Body Mass Index
DNA	deoxyribonucleic acid
FDR	false discovery rate
F	female
HbA1c	glycated hemoglobin
IQR	interquartile range
LEfSe	Linear discriminant analysis Effect Size
LDA	Linear Discriminant Analysis
M	male
Me	median
NGS	Next-generation sequencing
NMDS	Non-metric multidimensional scaling
OHI	Oral Hygiene Index
OTUs	Operational Taxonomic Units
PCR	Polymerase Chain Reaction
rRNA	ribosomal ribonucleic acid
SD	standard deviation
